# Delayed Presentation of Acute Tonsillitis Due to Monkeypox in an Immunocompromised Patient: Unique Cause of a Common Disease

**DOI:** 10.7759/cureus.43930

**Published:** 2023-08-22

**Authors:** Yusur Alsalihi, Likitha Y Aradhyula, Benjamin Teitelbaum

**Affiliations:** 1 Medicine, California Health Sciences University, Clovis, USA; 2 Medicine, California Health Sciences University, College of Osteopathic Medicine, Clovis, USA; 3 Surgery, Clovis Community Medical Center, Clovis, USA

**Keywords:** immunocompromised, tonsillitis, human immunodeficiency virus (hiv), human immunodeficiency virus, monkeypox virus rash, monkeypox, monkeypox virus

## Abstract

Monkeypox (Mpox) is a viral zoonotic disease caused by the Mpox virus, part of the *Orthopoxvirus* (OPXV) genus, which is a family of double-stranded DNA viruses. It typically presents with lymphadenopathy, fever, and most distinguishably, a rash that extends the cheeks, palms of hands, and soles of feet. This case report describes a unique manifestation of Mpox in a 71-year-old man with a past medical history of HIV, who presented to the emergency department with severe throat pain and oropharyngeal symptoms. Initially, the viral panel and blood cultures were negative, and the patient’s condition continued to deteriorate. Under clinical suspicion of Mpox, the patient was tested and found positive. This case report highlights the importance of vigilant surveillance and consideration of Mpox in immunocompromised patients with atypical presentations.

## Introduction

Monkeypox (Mpox) is a viral zoonotic disease that is caused by the Mpox virus, part of the *Orthopoxvirus* (OPXV) genus, which is a family of double-stranded DNA viruses [1]. Mpox has been noted as an increasing public health threat. The highest rates of Mpox are seen in regions of West Africa, where there is close contact between humans and wild animal reservoirs [2]. Diagnosis requires viral cultures and polymerase chain reaction (PCR) tests, and case fatality rates have ranged from 0% to 11% [1]. While the fatality rate of Mpox is smaller compared to that of smallpox, it can cause severe complications in those who are immunocompromised, such as pneumonia and encephalitis [3].

Symptoms of Mpox generally last two to four weeks, with an incubation period of around six to 13 days. The first five days of infection are called the invasion period, which manifests as lymphadenopathy, fever, back pain, and intense asthenia [1]. Patients will present with Mpox rash on the cheeks, palms of hands, and soles of feet. However, there is limited research and knowledge regarding Mpox presentation in the nose and oral cavity, especially regarding its involvement in the delayed nature of oropharyngeal disease. In this case report, we discuss a case of acute tonsillitis in an immunocompromised patient and review the current literature understanding of oropharyngeal involvement, along with the disease timeline of Mpox infection.

## Case presentation

A 71-year-old male patient with a past medical history of HIV infection presented to the emergency department with severe throat pain that has worsened over five days. The patient had no travel history and no history of exposure except for an unprotected sexual encounter within the past month. The patient did not receive his Mpox vaccine. The patient initially presented to his primary care and was prescribed amoxicillin under suspicion of strep A infection. He was three days into his course with no symptom resolution. The patient endorsed fever, nausea, vomiting, hoarse voice, and difficulty swallowing. Past medical history included HIV with a stable viral load, with CD4 cell count within the normal range of 500-1500 cells/mm^3, along with type 2 diabetes mellitus, hypertension, hyperlipidemia, and gastroesophageal reflux disease. The patient had hiatal hernia repair, and Nissen fundoplication was performed more than a year ago with no known complications. The patient consumes alcohol and denies any tobacco or drug use. Medications include Biktarvy (bictegravir/emtricitabine/tenofovir alafenamide) and ezetimibe since his diagnosis of HIV.

**Figure 1:**
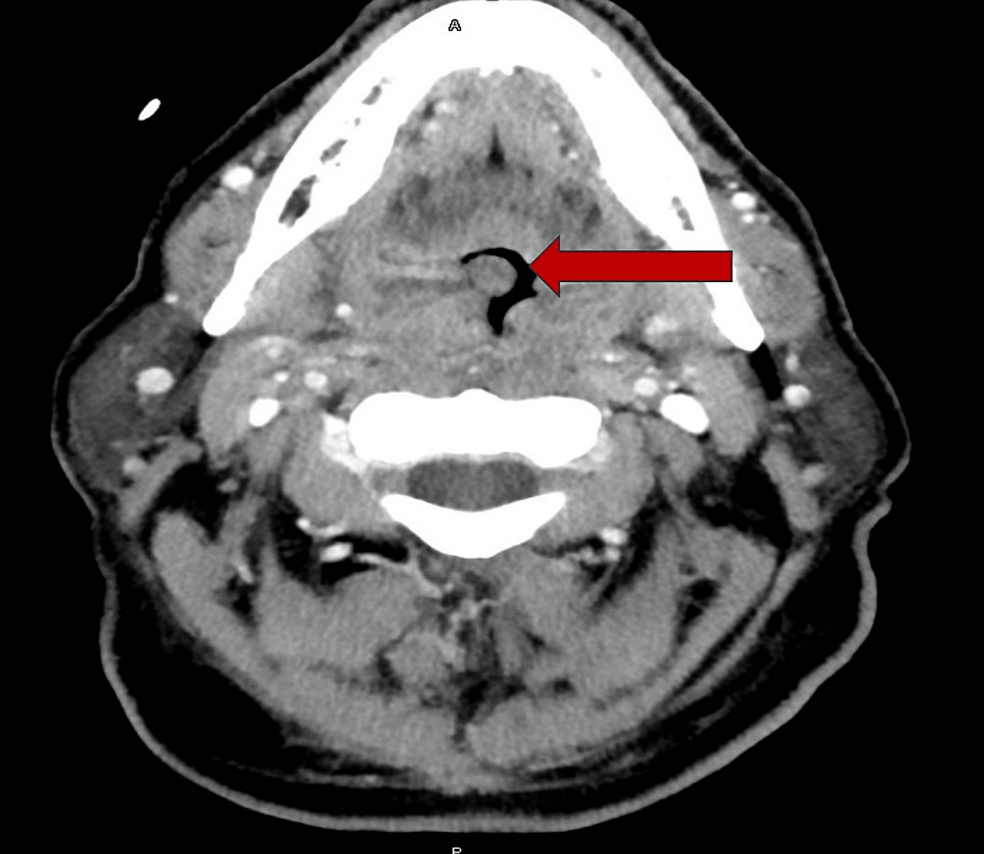
Axial view showing the enlargement of the tonsils.

**Figure 2:**
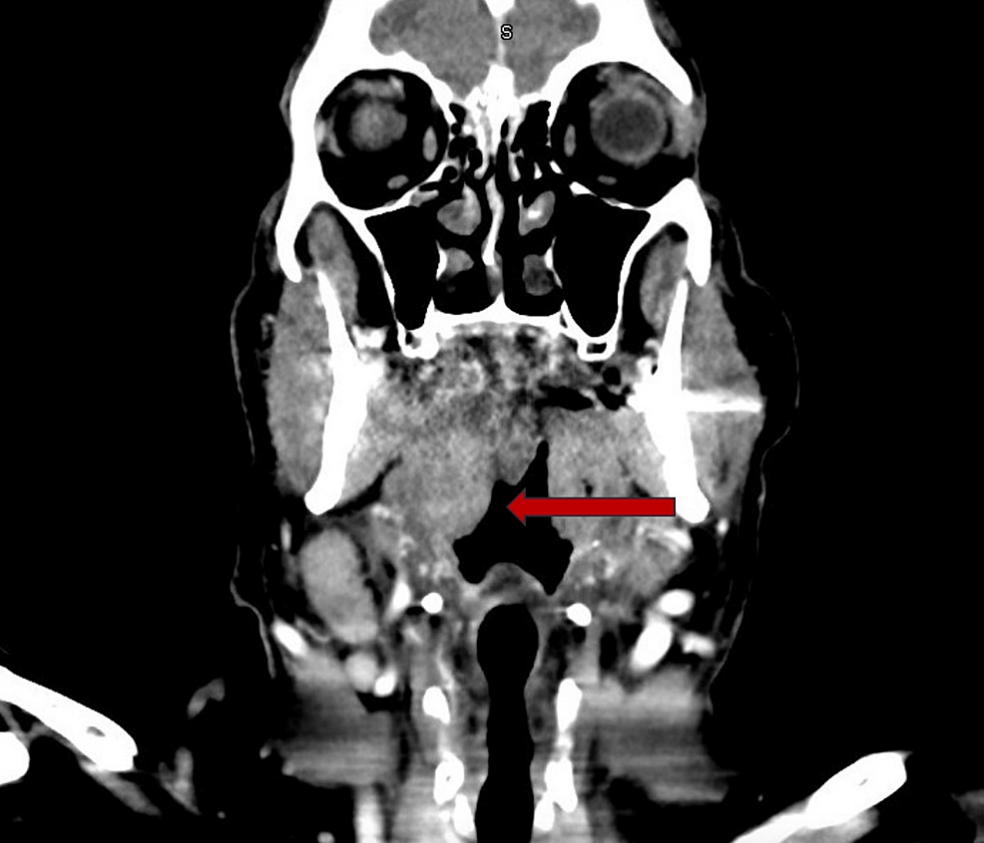
Coronal view showing reactive mucosal thickening and clear sinuses.

Vital signs included blood pressure of 144/72 mmHg, heart rate of 69 beats per minute, temperature of 98.6°F, respiratory rate of 18 breaths per minute, and SpO2 of 93% on room air. A physical exam showed multiple ulcers on the tongue, bilateral tonsillar hypertrophy and erythema with confluent exudates, soft palate erythema with minimal exudate, and bilateral zone 2 adenopathy. The patient initially underwent a CT of the neck with contrast, which showed an enlargement of palatine tonsils with areas of hypoattenuation and a possible small abscess measuring 0.5 cm bilaterally (Figure 1). The imaging also revealed a narrowing of the oropharyngeal airway (Figure 2). The results correlated with an infectious etiology, which made us explore viral and bacterial causes. A sexually transmitted diseases (STD) panel was negative. A viral respiratory panel, including replication-competent retrovirus (RCR), respiratory syncytial virus (RSV), human metapneumovirus, adenovirus, rhinovirus, and influenza A and B, was negative. Group A streptococcus and conventional blood cultures collected using venipuncture were negative after 72 hours.

The continued negative findings made us suspicious of malignancy. However, the lack of other symptoms, such as night sweats and weight loss, and the acute onset of presentation swayed away from malignancy. Furthermore, the presence of elevated white blood cells at the time of presentation (16.5 K/uL of blood) correlated with an infectious process.

Initial suspicion of Mpox came after all drug panels and bacterial cultures were negative. At this time, Mpox was at its height during the international Mpox outbreak of 2022, encouraging clinical suspicion. Upon further questioning the patient, he revealed that five days prior to presenting to the ED for the sore throat, he had pustular lesions on the dorsum of his hands that disappeared after 48 hours. He stated that he had complete resolutions of his symptoms and subsequently developed the sore throat and thus thought the two events were unrelated. PCR viral testing for Mpox was positive, and the patient was given the investigational drug tecovirimat 600 mg, which reported marked improvement. He was subsequently discharged three days later with a complete resolution of his symptoms.

## Discussion

The presented case offers valuable insights into the evolving nature of Mpox as a viral zoonotic disease and its emerging public health threat, particularly during the 2022 international outbreak. The patient's immunocompromised state due to HIV infection underscores the importance of vigilant surveillance and consideration of Mpox in the differential diagnosis of individuals with atypical presentations, such as acute tonsillitis. In an international clinical series study of the disease that included 528 cases across 16 countries, most cases in the United States came from men who have sex with men, the majority of which were white. Immunocompromised patients made up 41% of the reported cases [1].

Literature has also highlighted the most common clinical manifestations of the disease with 95% of the cases presenting with rash, and 64% of those presenting with a rash have less than 11 pustular lesions. The majority of the rashes were in the anogenital region (73%), which could be secondary to Mpox being transmitted via sexual contact [1]. The most common systemic signs of infection were fever (62%) and lethargy (41%) [1]. Diagnosing Mpox, given its clinical resemblance to other infectious diseases, poses diagnostic challenges. Clinical suspicion, informed by epidemiological factors, is paramount, especially during outbreaks. The positive viral testing for Mpox utilizes viral culture and PCR techniques played a decisive role in confirming the diagnosis and highlights the significance of appropriate diagnostic methodologies for timely and accurate identification.

This atypical presentation and lack of dermatological symptoms could be due to multiple factors. Due to the immunocompromised nature of the patient, he could not mount a significant response to Mpox. Thus, this might have alluded to the lack of neutrophil-filled pustule presentation the disease is known for. Another factor could be due to a dermatological presentation and resolution before presenting to the ED. A viral mutation in the Mpox strain could explain the lack of pustule presentation and fever in Mpox-infected patients.

Oropharyngeal symptoms have been a less common presentation of Mpox with a meta-analysis reporting the incidence of oropharyngeal symptoms in Mpox patients to be approximately 37% [4]. The most common clinical finding in oropharyngeal disease presentation is sore throat (40%), followed by mouth sore (25%) and tonsillitis (18%). This patient had a presentation of tonsillitis and mouth ulcers correlating with the most common clinical findings.

Our patient had a rash on his hands a week prior to the ED presentation for tonsillitis; however, he did not correlate the two infections to the same causative agent. This could highlight a challenge for clinicians as they try to discern a thorough medical history, as often patients may not be aware of the presence of the rash. This delayed presentation has not been reported in the literature to the knowledge of the authors of the paper. According to the CDC, in some recent Mpox cases, people have presented with a rash without a recognized prodrome [5]. However, no research has shown that oropharyngeal disease was the delayed clinical presentation following the resolution of the initial rash.

## Conclusions

In conclusion, this case report offers valuable insights into the evolving nature of Mpox and its implications for public health. Clinicians should not be deterred by the lack of a rash or other classical manifestations of the disease. The presented case underscores the importance of considering Mpox in the differential diagnosis, particularly in immunocompromised patients with atypical presentations of oropharyngeal disease and negative standard viral and bacterial cultures. As oropharyngeal symptoms may be less common in Mpox, the report reinforces the need for heightened awareness of potential variations in clinical manifestations.

Further research and surveillance efforts are warranted to enhance our understanding of Mpox and improve diagnostic capabilities, ultimately enabling better preparedness and management strategies to tackle this emerging public health concern. With Mpox posing an increasing public health threat, clinicians and healthcare providers should remain vigilant, especially during outbreaks, to promptly identify and manage cases.
